# Post-ovulatory Hemoperitoneum in a Patient on Anticoagulation Therapy for Mitral Valve Replacement

**DOI:** 10.7759/cureus.33674

**Published:** 2023-01-12

**Authors:** Shaikh Muneeba, Neema Acharya, Sourya Acharya, Ketav Joshi, Shazia Mohammad

**Affiliations:** 1 Department of Obstetrics and Gynaecology, Jawaharlal Nehru Medical College, Datta Meghe Institute of Higher Education & Research, Wardha, IND; 2 Department of Medicine, Jawaharlal Nehru Medical College, Datta Meghe Institute of Higher Education & Research, Wardha, IND

**Keywords:** post-ovulation, pt-inr, anticoagulation therapy, warfarin, hemoperitoneum, corpus luteal cyst

## Abstract

When a patient is receiving anticoagulant therapy, the rupture of a corpus luteum cyst may go unrecognized in healthy women but becomes clinically relevant as it might exacerbate a hemoperitoneum episode. This report describes the case of a 26-year-old primipara who underwent surgical treatment for a heart defect and later experienced extensive hemoperitoneum. The patient reported to the casualty with symptoms of unstable hemodynamic status such as hypotension 90/60 mmHg and tachycardia 120 beats/minute. A multidisciplinary team decided upon surgical management after stabilizing the coagulation profile and correcting the shock with blood and blood products. The reason was discovered to be a ruptured cyst wall, which was fixed electrosurgically. The patient had a full recovery with no postoperative complications. The most noteworthy aspect of this case was the catastrophic hemoperitoneum caused by improper anticoagulant treatment monitoring. Management of such cases depends on the age of the patient, fertility, and calculating the long-term prognosis of the anticoagulation therapy for the patient.

## Introduction

In women of the reproductive age group, hemoperitoneum and a ruptured corpus luteum cyst are common, as supported by the study of Ho et al., where a total of 91 women in the age group of 15-42 years with a mean age of 26 years were diagnosed with ruptured corpus luteum cyst and hemoperitoneum, and 60.4% of ruptured cysts were found during the secretory phase [1]. Differential diagnosis is typically a ruptured ectopic pregnancy. However, a rare consequence in women using anticoagulants is a ruptured corpus luteum with hemoperitoneum. It can sometimes reveal itself in a big way that necessitates surgery and blood transfusions. Anticoagulant-treated patients are more likely to experience major life-threatening hemorrhages from ruptured corpus luteum [2]. Therefore, for optimal care, an accurate diagnosis based on history, clinical examination, and investigation is required.

## Case presentation

A 26-year-old primipara presented to the casualty with complaints of pain in the abdomen in the right iliac region for two days. The patient complained of abdominal pain, which gradually progressed to excruciating pain, increasing with changes in position. The patient was a known case of mitral valve regurgitation and had undergone surgery for mitral valve replacement seven years ago. After the mitral valve replacement, the patient was started on anticoagulation medications tablet warfarin (6 mg at 6 pm/daily), tablet furosemide (40 mg once daily), tablet aspirin (75 mg once daily), tab digoxin (0.25 mg once daily), injection benzathine penicillin 12 lac IU intramuscularly every 21 days. However, proper documents and reports were not maintained by the patient. On presentation, she was peri-ovulatory with regular monthly cycles. Neither bleeding disorders nor a family history of orifice-related bleeding was present. On general examination, the patient looked anxious and showed signs of hypovolemic shock with hypotension, tachycardia, and raised jugular venous pressure. The respiratory examination was normal with air entry equal on both sides, no basal creeps were heard, and oxygen saturation was normal. On cardiovascular examination, S1 and S2 were heard with a metallic click and a loud P2. No abnormality was detected on the central nervous system examination. On abdominal examination, there was distinct distension with marked tenderness in the iliac fossae and hypogastrium with rebound tenderness. There was no palpable mass. The liver and spleen were not palpable.

**Figure 1:**
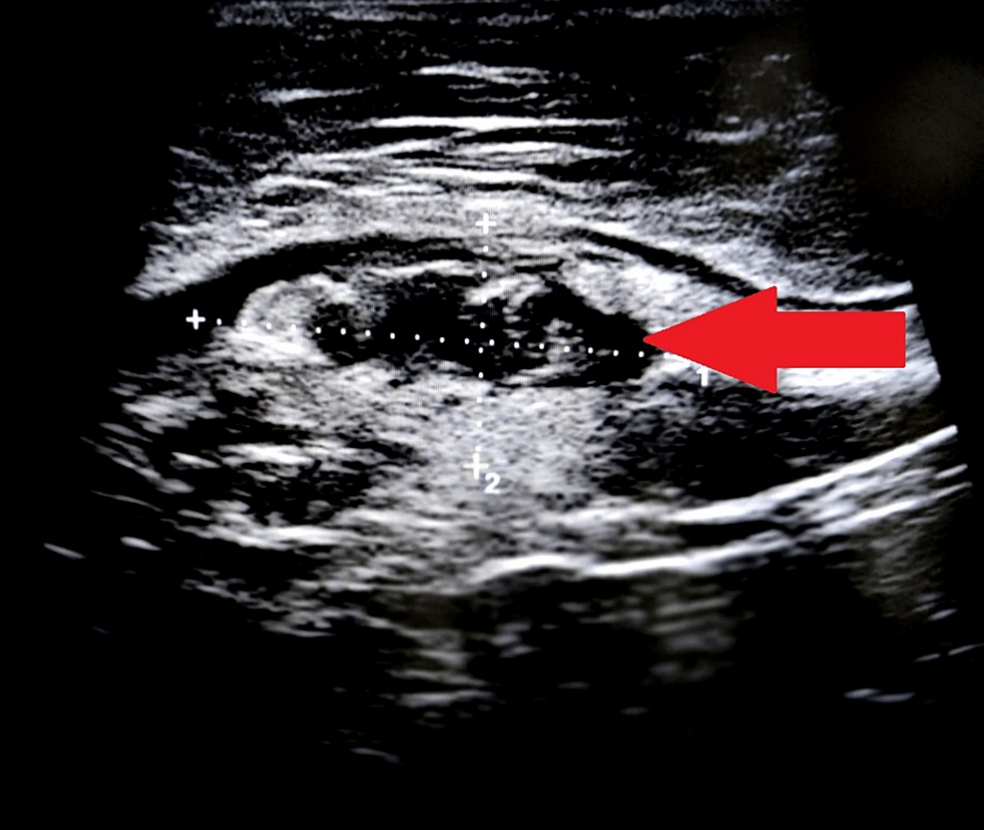
Ultrasound image showing peripheral compound cyst with a few septa.

**Figure 2:**
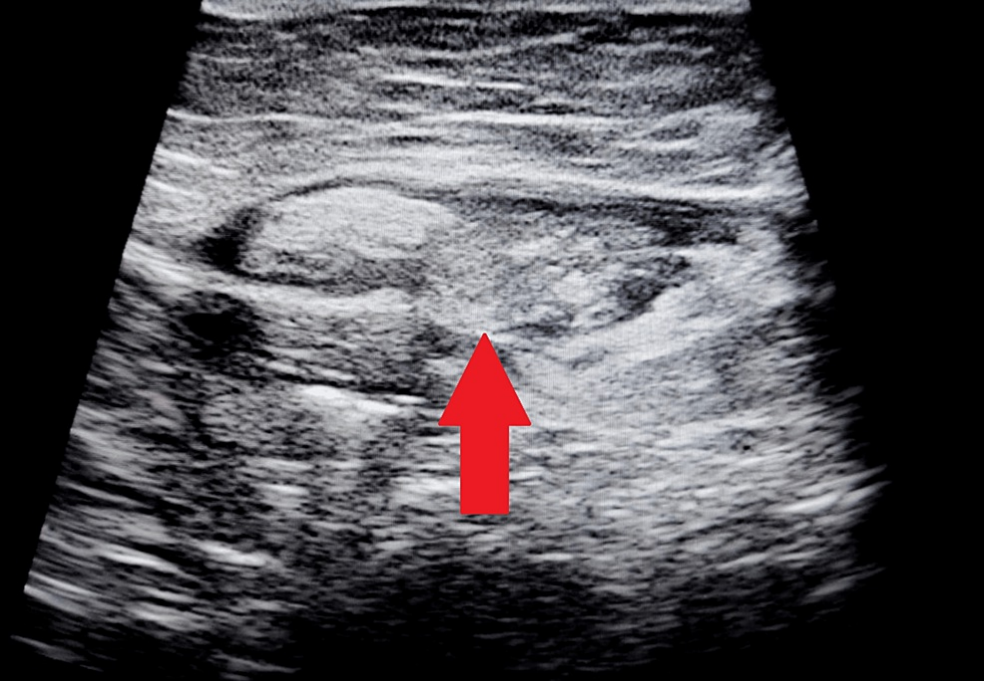
Ultrasound image showing hemoperitoneum.

On per vaginum examination, the cervix was downwards backward, and the uterus was anteverted, anteflexed, normal in size, and mobile. There was tenderness in all fornices. Her menstrual cycle was for about three to four days every 30 days, with regular average flow associated with no clots and no dysmenorrhea. On presentation, she was peri-ovulatory. Her blood profile showed severe anemia with normocytic normochromic features on the peripheral smear. The coagulation profile of the patient revealed a prothrombin time (PT) of 56 seconds with an international normalized ratio (INR) of 5.28 and a partial thromboplastin time (PTT) of 57 seconds. A urine pregnancy test and a human chorionic gonadotropin level were performed to rule out pregnancy. Tests for the function of the liver and kidneys were within normal parameters, and cancer antigen-125 levels were 364 U/mL. Ultrasound of the abdomen revealed an ill-defined right heterogeneous hyperechoic mass showing a peripheral compound cyst with few septa of size 4.5 cm × 3.5 cm mass with no increased vascularity on Doppler, suggestive of a ruptured hemorrhagic ovarian cyst (Figures 1, 2).

**Figure 3:**
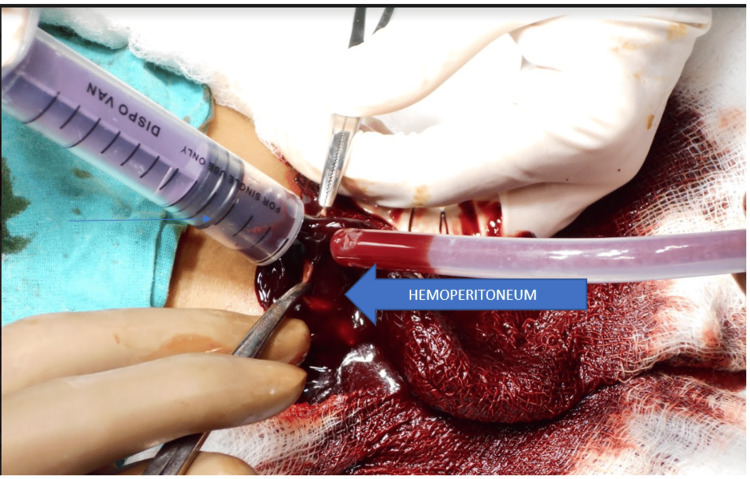
Intraoperative image showing hemoperitoneum.

A two-dimensional (2D) echocardiography showed the prosthetic valve functioning well with the AR jet hitting the interventricular system at the peri membranous area giving rise to a diastolic movement of the interventricular septum (IVS) toward the left ventricle causing outpouching of IVS, mild tricuspid regurgitation, and mild-to-moderate mitral valve regurgitation with mild pulmonary hypertension in sinus rhythm. Ectopic pregnancy was one of the possible differential diagnoses in this case, but a urine pregnancy test ruled it out. All of the aforementioned observations and tests concluded that anticoagulant therapy-induced hemoperitoneum resulted from a functional ovarian cyst rupture (Figure 3).

The patient was not compliant and did not maintain any follow-up investigations after the mitral valve replacement. The last blood investigations of the patient before the hemoperitoneum episode were found to be seven months back which revealed a normal coagulation profile. Because the patient’s overall health did not call for surgical exploration, a multidisciplinary team of doctors, including cardiologists and gynecologists, agreed to pursue a conservative course of treatment. With advice from the cardiothoracic surgery team, the dosage of anticoagulants was changed and tapered down. The patient was put on low-molecular-weight heparin 0.6 mL S/C BD. The surgical approach was deferred till PT-INR normalized. Tranexamic acid (3 g/day intravenously in divided doses) was started. The patient gradually improved after receiving four fresh-frozen plasma (FFP) units and three packed red cell (PRC) units. However, despite conservative management on day three of admission, the patient’s vitals did not improve.

An ultrasound of the pelvis was done to review the status of the hemoperitoneum, which revealed the same amount of hemoperitoneum with a mild increase in the size of the adnexal cyst. The decision of exploratory laparotomy was undertaken as the patient’s general condition was not fit for a laparoscopic approach. Explorative laparotomy was undertaken by minilap laparotomy incision, which revealed approximately 1,400 mL of hemoperitoneum, with 500 g or more clots in the pouch of Douglas. Active bleeding from the cyst wall diagnosed a right-sided ruptured ovarian cyst. Both sides had normal-looking ovarian tissues. Electrosurgery was used to remove the cyst wall. The bleeding cyst was sutured with an absorbable suture. The visceral organs were inspected during the operation, and no signs of hemorrhage, endometriosis, or adhesions were found. The peritoneal and urogenital surfaces were unremarkable. During the procedure, two units of PRC and four units of FFP were transfused. By locating the bleeding cause and draining the peritoneum, hemostasis was attained during the procedure.

Because the patient was in the reproductive stage and wished for future conception, the management of the patient was done with the conservation of fertility despite using surgical methods. The incised tissue was taken for histopathological examination, where the corpus luteum wall appeared normal. After being stained with hematoxylin and eosin, the tissue from the incised cyst wall was studied under a microscope, revealing that the luteal cyst wall had a well-organized thrombus with patches of red blood cells, surrounding fibrin accumulations, and dispersed leucocytes. The patient was given an injection leuprolide acetate 3.75 mg intramuscularly for ovulation suppression till further reproduction was planned. Following a recommendation for a coagulation profile after 15 days, monthly monitoring was to continue until an INR of 2.5 was considered therapeutically acceptable. Thereafter, thorough three-monthly monitoring was to be carried out. The couple was counseled for periconceptional multidisciplinary counseling and planned pregnancy and to use a barrier method of contraception till then.

## Discussion

Ovarian cysts typically occur in menstruating females aged 18-35 years and are commonly determined by ultrasound. Two kinds of practical ovarian cysts are described, namely, follicular cysts and corpus luteum cysts. The corpus luteum is an extremely vascular framework. The rate of bloodstream to the corpus luteum surpasses any adult organ (each tissue). The enhanced bloodstream provides substrates for hormonal agent manufacturing and supports the quick splitting of luteal cells [3]. Corpus luteum cysts are thin-walled, practical vascular frameworks, most of which are predisposed to rupture. The tear of the corpus luteum cyst is just one of the significant gynecologic reasons for hemoperitoneum. The etiology of cyst tears is unknown, although it has been recommended that the enhanced vascularity of the ovary in the luteal stage and maternity might predispose to a corpus luteal cyst rupture. There are two groups of women who are more susceptible to corpus luteum hemorrhage: first, those who are taking anticoagulants due to a thrombotic disorder, and, second, those who have congenital bleeding disorders [4].

The INR value of 3.0-4.5, with levels above 4.5, also significantly increases the risk of hemorrhage [5]. Corpus luteal cysts develop due to the corpus luteum’s rapid development and strong vascularity, which cause intraluteal hemorrhage and the growth of a hemorrhagic cyst [6]. Most reported cases of ruptured cysts have occurred in the secretory phase and onset of pain after recent sexual activity [1]. A cyst bleeding continuously may quickly expand, spontaneously tear, or seep blood into the peritoneal cavity. After ovulation, a stage of proliferation or hyperemia includes follicular collapse and luteinization of the bloodless granulosa layer [7]. Blood is still absent from the corpus luteum’s lumen. The granulosa layer is then penetrated by blood vessels, which fill the cavity of the corpus luteum with blood, signaling the stage of vascularization. The inhibition of the coagulation cascade in anticoagulant medication patients makes them more susceptible to intraperitoneal hemorrhage and uncontrolled bleeding from corpus cyst wall hematoma rupture.

Women of reproductive age receiving anticoagulant medication should be aware of the possibility of hemorrhage from a burst corpus luteum cyst because it is a potentially fatal adverse effect [7]. Anticoagulant medications are tailored according to the INR of the patient. Corpus luteum rupture should always be suspected in women who have known bleeding issues or are taking anticoagulant medication if they experience lower quadrant stomach pain. For the early detection of patients with bleeding disorders, comprehensive coagulation screening is necessary; anamnesis, anticoagulant medication, and family history can provide useful information [7]. These patients have a higher chance of developing recurrent HCL, so ovulation should be controlled using low-dose oral contraceptive pills to avoid reoccurring instances. They prevent ovulation and the subsequent development of the corpus luteum [8], progesterone-only medications where follow-up should include general and gynecological examination, lipid profile [9], the hormonal intrauterine device with levonorgestrel which acts for a long duration of seven years and reduces the menstrual blood loss [10], or analogs of the gonadotropin-releasing hormone. For the management of pain, non-steroidal anti-inflammatory drugs are the drug of choice. Still, they should be used mindfully by estimating the clinical risk associated with drug interaction with anticoagulant medications or associated liver diseases [11].

Joshi et al. (2022) reported a similar case of hemoperitoneum due to a ruptured corpus luteal cyst secondary to a coagulation defect which was managed by exploratory laparotomy with electrosurgical excision of the cyst wall [3]. Agarwal et al. (2017) India managed the case of a ruptured corpus luteum cyst with hemoperitoneum due to unmonitored warfarin therapy post-mitral valve replacement by conservative methods [12]. Ara et al. (2016) reported a case series of ruptured complex ovarian cysts that presented with acute abdomen with hemoperitoneum secondary to complex ovarian cyst hemorrhage with etiology of long-term warfarin post-mitral valve replacement, both cases were managed conservatively [13]. Sikka et al. (2015) conservatively managed the case of complex left ovarian cyst hemorrhage with acute abdomen and gross peritoneal free fluid due to long-term warfarin therapy post-mitral valve replacement surgery [14]. Centinkaya et al. (2011) managed a case of peri-ovulatory bleeding with hemoperitoneum with underlying etiology of congenital afibrinogenemia by exploratory laparotomy with cystectomy and evacuation of hemoperitoneum, and the second episode of hemoperitoneum was managed conservatively [15].

## Conclusions

Ovulation-related bleeding and hemoperitoneum are rare but known complications. It is nearly fatal in cases of unmonitored anticoagulation therapy such as in patients who are put on oral anticoagulants for prosthetic heart valves. These patients need strict and vigilant monitoring of the coagulation profile and titration of dosage of anticoagulation medications. The mode of management of such a clinical scenario should depend upon the amount of hemorrhage and the hemodynamic status of the patient. In mild cases, conservative management should be the first mode, while in severe cases, after correcting the coagulation profile surgical exploration may be needed. If compliance is an issue for monitoring of coagulation profile, such young women in the reproductive age group receiving anticoagulation therapy should be counseled and treated with ovarian suppression therapy, thereby preventing related hemorrhage. All young women needing anticoagulation therapy should be managed in a multidisciplinary manner involving a gynecologist or a reproductive medicine specialist to avoid such near-fatal conditions. Surgical and medical methods of treatment should be individualized depending on the risks and benefits ratio of each individual while keeping in mind the long-term complications of the therapy for the patient.
